# Human action recognition based on HOIRM feature fusion and AP clustering BOW

**DOI:** 10.1371/journal.pone.0219910

**Published:** 2019-07-25

**Authors:** Ruo-Hong Huan, Chao-Jie Xie, Feng Guo, Kai-Kai Chi, Ke-Ji Mao, Ying-Long Li, Yun Pan

**Affiliations:** 1 College of Computer Science and Technology, Zhejiang University of Technology, Hangzhou, Zhejiang, China; 2 Department of Information Science and Electronic Engineering, Zhejiang University, Hangzhou, Zhejiang, China; Hangzhou Normal University, CHINA

## Abstract

In this paper, we propose a human action recognition method using HOIRM (histogram of oriented interest region motion) feature fusion and a BOW (bag of words) model based on AP (affinity propagation) clustering. First, a HOIRM feature extraction method based on spatiotemporal interest points ROI is proposed. HOIRM can be regarded as a middle-level feature between local and global features. Then, HOIRM is fused with 3D HOG and 3D HOF local features using a cumulative histogram. The method further improves the robustness of local features to camera view angle and distance variations in complex scenes, which in turn improves the correct rate of action recognition. Finally, a BOW model based on AP clustering is proposed and applied to action classification. It obtains the appropriate visual dictionary capacity and achieves better clustering effect for the joint description of a variety of features. The experimental results demonstrate that by using the fused features with the proposed BOW model, the average recognition rate is 95.75% in the KTH database, and 88.25% in the UCF database, which are both higher than those by using only 3D HOG+3D HOF or HOIRM features. Moreover, the average recognition rate achieved by the proposed method in the two databases is higher than that obtained by other methods.

## Introduction

The aim of video-based human action recognition is to recognize human action patterns in video; it has wide application in intelligent monitoring, human–computer interaction, and video searching [1]. Human action recognition mainly includes two steps: feature extraction and description, and action classification and recognition.

Regarding the extraction and description of action features, video-based human action recognition methods are based either on global features or on local spatiotemporal interest points. The former have become the mainstream owing to their robustness to various types of interference. These methods extract the underlying features for action description by detecting interest points using significant pixel value changes in the spatiotemporal neighborhood, without requiring object detection, image segmentation, and target tracking. However, local features are decentralized, and the global properties of the human body are ignored. Thus, when local-feature-based methods are tested on video databases where human body contours are simple, the recognition rate is even slightly lower than that of global-feature-based methods [2]. Accordingly, direct fusion of global and local features has been proposed to improve the recognition rate. To preserve the performance of the local descriptor and the overall distribution information globally in space and time, Ji et al. [3] proposed a novel method for representing human motion by combining local and global information. Specifically, they combined a 3D SIFT descriptor and spatiotemporal distribution information based on interest points. Bregonzio et al. [4] used COP (clouds of points) and BOW (bag of words) features extracted from spatiotemporal interest points, which are easy to compute and robust against noise and occlusion. The proposed COP representation at multiple temporal scales exploits the distribution explicitly, capturing both local and global temporal information. Lin et al. [5] proposed a framework for transferring action models in human action recognition. They extracted local spatiotemporal interest point features and global shape–flow features as low-level features, and constructed a hybrid BOW model using the corresponding view-specific codebook for each action sequence. Action models can be directly transferred across views, and this framework proved effective in experiments. Ryoo et al. [6] recognized interaction-level human activities using local and global motion features. They extracted local and global motion features; moreover, they described multi-channel kernels and combined them for recognition.

Even though the recognition rate has been improved in the above studies, the two feature categories are difficult to fuse, and the extraction of global features is highly complicated, which always includes object detection, image segmentation, and target tracking. In this paper, a HOIRM (histogram of oriented interest region motion) feature extraction method is proposed. The extracted HOIRM features can be regarded as middle-level features between local and global features. They combine the advantages of both local and global features and avoid the complicated steps required for global feature extraction. Then, we fuse the HOIRM features with 3D HOG (histogram of oriented gradients) and 3D HOF (histogram of oriented optical flow) local features using a cumulative histogram to describe human action. This improves the robustness of local features to camera view angle and distance variations in complex scenes, which in turn improves the correct rate of action recognition.

In the classification and recognition stage, methods based on local spatiotemporal interest points generally use BOW to model and classify actions. To improve the action recognition rate, several local-feature-based action recognition methods use a variety of spatiotemporal interest point descriptors to describe actions in the feature extraction stage. Thus, the number of the extracted local spatiotemporal features is often large, and therefore when the BOW model is applied to video processing, it is impossible to regard all the descriptive vectors as vocabularies. Generally, some descriptive vectors are regarded as vocabularies. The usual practice is to cluster all the descriptive sub-vectors by K-Means clustering, and the center of each resulting cluster is regarded as a vocabulary. To recognize an aggressive human action, such as boxing, Ouanane et al. [7, 8] used an offline K-Means algorithm to assign an appropriate label to each previously obtained low-feature vector. The number of clusters is fixed a priori and depends on the period of the boxing actions. To detect crowd actions, Pathan et al. [9] treated the flow field in each block-clip as a 2D sample distribution and used a Gaussian mixture to maintain the generality of the flow field. Then, they used K-Means clustering to initialize and find clusters in this distribution. Elshourbagy et al. [10] used multilevel K-Means for human activity recognition. Compared to the K-Means algorithm, the multilevel K-Means algorithm increases computational complexity and reduces memory usage.

When the BOW model is used with K-Means clustering, multiple tests are required to obtain better dictionary capacity and improve the recognition rate. It is difficult to confirm whether the dictionary capacity is optimal, and it is also worth considering whether the K-Means clustering algorithm itself is best for clustering a variety of features in the form of a joint description. In this paper, a BOW model based on AP clustering is proposed. After multi-feature fusion, the AP clustering BOW algorithm is used to construct a visual dictionary. Compared with the BOW model based on the K-Means clustering algorithm, the proposed BOW model does not require multiple experiments but only one to obtain the appropriate visual dictionary capacity. Moreover, it has a better clustering effect on the joint description of a variety of features, which improves the recognition rate.

The contributions of this study are as follows: (1) A HOIRM feature extraction method is proposed. The extracted HOIRM features can be regarded as middle-level features between local and global features. They have the advantages of both local and global features and avoid the complicated steps required for global feature extraction. (2) HOIRM feature is fused with 3D HOG and 3D HOF local features using a cumulative histogram to describe human action. It improvs the robustness of local features to camera view angle and distance variations in complex scenes, which in turn improves the correct rate of action recognition. (3) A BOW model based on AP clustering is proposed for action recognition. Compared with the BOW model based on the K-Means clustering algorithm, the proposed does not require multiple experiments but only one to obtain the appropriate visual dictionary capacity. Moreover, it has a better clustering effect on the joint description of a variety of features, which improves the recognition rate. The experimental results demonstrate that by using the fused features with the proposed BOW model, the average recognition rate is 95.75% in the KTH database, and 88.25% in the UCF database, which are both higher than that by using only 3D HOG+3D HOF or HOIRM features. Furthermore, the average recognition rate achieved by the proposed method in these databases is higher than that obtained by other methods.

This paper is organized as follows: Section 2 presents related work. The HOIRM feature extraction method is presented in Section 3. Multiple feature fusion is described in Section 4. The BOW model based on AP clustering is described in Section 5. Section 6 presents the experimental results and analysis. Section 7 concludes the paper.

## Related work

Naidoo et al. [11] proposed a method that generates motion history images and extracts features using the bag of features approach for training. This approach extracts speed up robust features and then clusters them using K-Means clustering to form a training vector. The method can achieve an accuracy of 82.00% on the KTH dataset. Jaouedi et al. [12] proposed a method that uses human subtraction analysis by a Gaussian mixture model and body movement analysis through trajectory models constructed from Kalman filters. These models remove noise by extracting the main motion features and constitute a stable base to identify the evolution of human activity; they can achieve an accuracy of 91.00% on the KTH dataset. Zhang et al. [13] developed a novel 3D CNN (convolutional neural network) model for action recognition instead of the traditional 2D inputs. It extracts features that contain more information and achieves an accuracy of 91.67% on the KTH dataset. Laptev et al. [14] detected interest points using a space–time extension of the Harris operator and a multi-scale approach, and extracted features at multiple spatiotemporal scales to characterize local motion and appearance; it can achieve an accuracy of 91.80% on the KTH dataset. Najar et al. [15] developed a novel learning algorithm based on the fixed-point covariance matrix estimator combined with the expectation–maximization algorithm and proposed an appropriate minimum message length criterion for model selection; the method can achieve an accuracy of 91.97% on the KTH dataset. Yuan et al. [16] proposed a discriminative pattern matching method called naive-Bayes-based mutual information maximization for multi-class action categorization and proposed a novel search algorithm to locate the optimal subvolume in the 3D video space for efficient action detection. It can handle well intra-pattern action variations, such as scale and speed variations; moreover, it is insensitive to dynamic and clutter backgrounds and even partial occlusions; it achieves an accuracy of 93.30% on the KTH dataset. Tong et al. [17] presented a new nonnegative matrix factorization with local constraint and proposed a nonnegative matrix factorization with temporal dependencies constraint; the method can achieve an accuracy of 93.96% on the KTH dataset. Fu et al. [18] proposed a method that uses multi-scale volumetric video representation and adaptively selects an optimal space–time scale under which the saliency of a patch is the most significant; the method can achieve an accuracy of 94.33% on the KTH dataset. Kovashka et al. [19] proposed a method that first extracts local motion and appearance features, quantizes them to a visual vocabulary, and then forms candidate neighborhoods consisting of the words associated with nearby points and their orientation with respect to the central interest point; the method can achieve an accuracy of 94.53% on the KTH dataset. Wang et al. [20] proposed a method that combines a dense sampling detector and a HOG 3D descriptor; it can achieve an accuracy of 85.60% on the UCF dataset. Kläser et al. [21] evaluated an “external” use of human tracks by treating the tracks as an approximate actor-background segmentation to suppress clutter; moreover, they investigated an “internal” use of tracks by learning action models with stronger geometry; the method achieves an accuracy of 86.70% on the UCF dataset. Bregonzio et al. [22] proposed a new action representation method based on computing a rich set of descriptors from key point trajectories, and developed an adaptive feature fusion method to combine different local motion descriptors for improving model robustness against feature noise and background clutters. The method can achieve an accuracy of 86.90% on the UCF dataset. Farrajota et al. [23] proposed a method for handling low-level actions by combining human body joint information to aid action recognition. This is achieved by using high-level features computed by a CNN that is pre-trained on ImageNet, with articulated body joints as low-level features. These features are then used to feed a long-short-term memory network to learn the temporal dependencies of an action; the method achieves an accuracy of 87.20% on the UCF dataset.

## HOIRM feature extraction method

### Spatiotemporal interest points detection

Bregonzio et al. [24] proposed an effective interest point detection algorithm. Compared with the most commonly used Harris 3D detection algorithm [25] and the Dollar detection algorithm [26], the spatiotemporal interest points detected by the Bregonzio algorithm are more descriptive. Furthermore, their distribution is more concentrated in the body part of the moving area, which allows obtaining the region of interest (ROI) by considering the changes in this distribution. Thus, we chose the Bregonzio algorithm to detect the spatiotemporal interest points in the proposed method.

### Region of interest extraction

**Fig 1:**
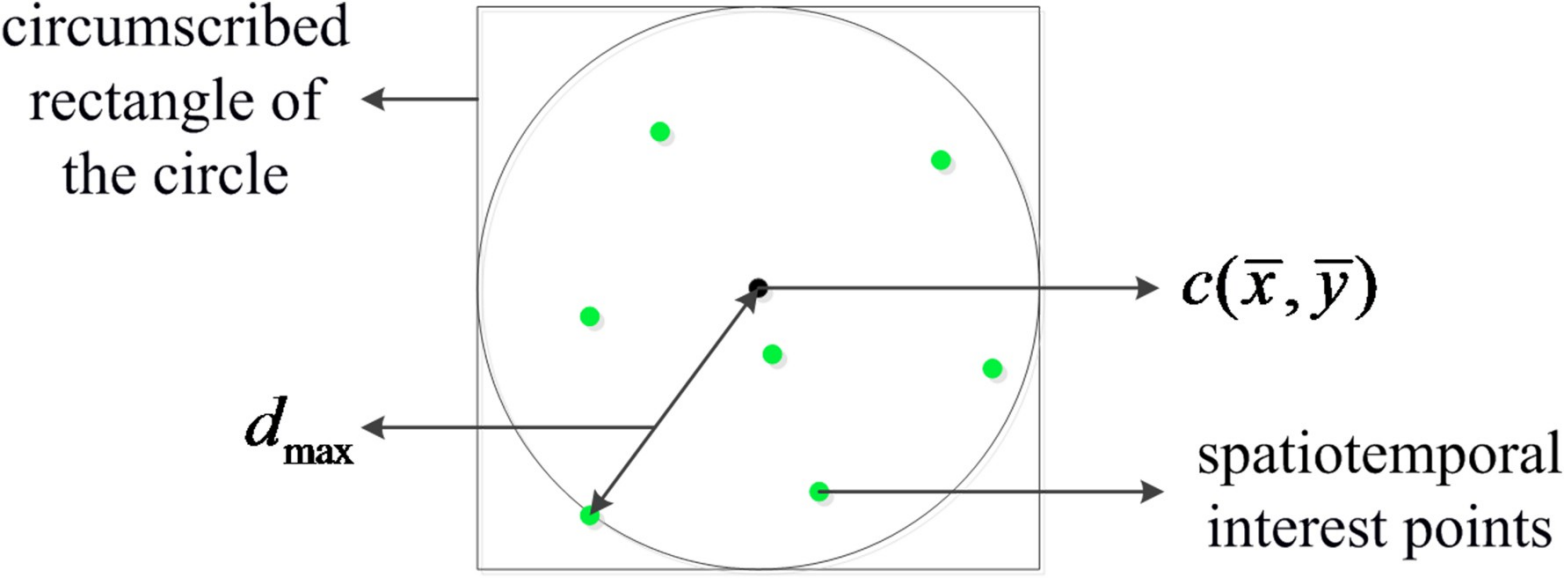
Determining the region of spatiotemporal interest points.

We determined the region of spatiotemporal interest points according to their distribution in each frame, as shown in Fig 1. The steps of ROI extraction are as follows:

1. Detect the Bregonzio spatiotemporal interest points and obtain their coordinates in each frame (green dots in Fig 1).
2. Calculate the centroid position $c(\bar{x},\bar{y})$ of all interest points in each frame, where $\bar{x}=\frac{1}{n}\sum_{i=1}^{n}x_{i}$, $\bar{y}=\frac{1}{n}\sum_{i=1}^{n}y_{i}$, *x*_*i*_ and *y*_*i*_ denote the horizontal and vertical coordinates of the i-th interest point, respectively, and *n* denotes the number of interest points in the current frame.
3. Calculate the distances *d*_*i*_ from all interest points to the centroid and select the maximum distance *d*_max_ = max{*d*_1_, *d*_2_, …, *d*_*n*_}.
4. Define a circle with center $c(\bar{x},\bar{y})$ and radius *d*_max_.
5. Using the centroid $c(\bar{x},\bar{y})$ as the center and the diameter of the circle as the side length, generate the circumscribed rectangle, which is the ROI in the current frame.

### HOIRM feature extraction

We chose the centroid of the ROI as a representative of the motion of the ROI so that the motion direction of each frame in the video may be accurately described. It follows that at any time *t*, the direction angle of the centroid of the ROI is

$$\theta ({\bar{x}}_{i},{\bar{y}}_{i},t)=\text{arctan}\frac{{\bar{y}}_{i}-{\bar{y}}_{\left(i-1\right)}}{{\bar{x}}_{i}-{\bar{x}}_{\left(i-1\right)}}.$$ (1)

The direction angle is quantified into *K* intervals according to HOG to facilitate the subsequent process of feature fusion. We set *K* = 9, that is, nine intervals are used. The interval size is 20° and the intervals are 0°–20°, 20°–40°,…, 140°–160°, and 160°–180°. The number of directions in each interval is counted by using Eq (1), and HOIRM is formed, which can be regarded as the motion trend of the ROI. HOIRM is the ratio of the number of frames in this direction angle interval to the total number of frames:

$$HOIRM\text{\%}=\frac{\text{NUM}(\theta ({\bar{x}}_{i},{\bar{y}}_{i},t)\in \theta_{i})}{\text{NUM}(frames)}\times 100\%.$$ (2)

**Fig 2:**
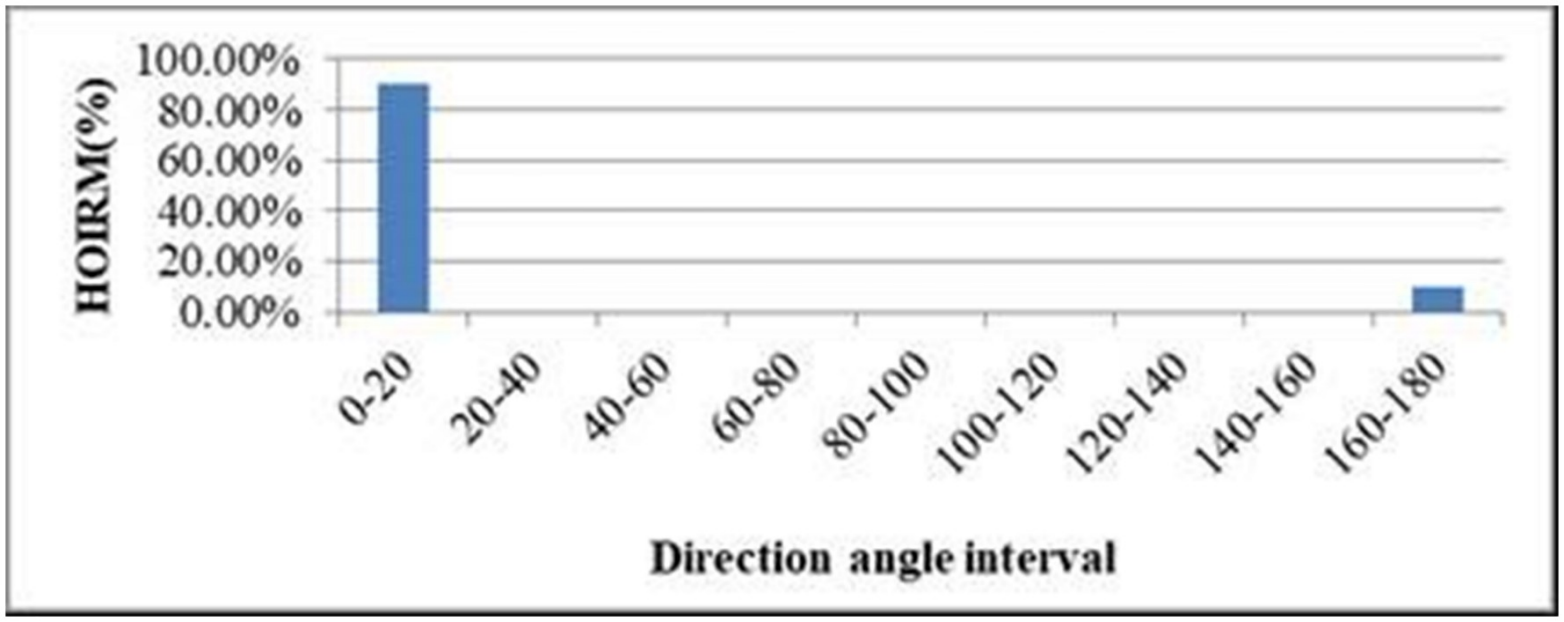
HOIRM of “waving”.

We picked a video containing “waving” action from KTH Human Action Database [27] and extracted regions of interest for some frames. Fig 2 shows the HOIRM for “waving”, in which the horizontal coordinates correspond to the intervals of the direction angle, and the vertical coordinates correspond to HOIRM. It can be seen that the motion direction angles of the ROI are less than 20° for most of the frames. When the gesture is up-down or down-up waving, the motion direction change is significant and the direction angle is close to 180°.

## Multiple feature fusion

### 3D HOG and 3D HOF feature description

**Fig 3:**
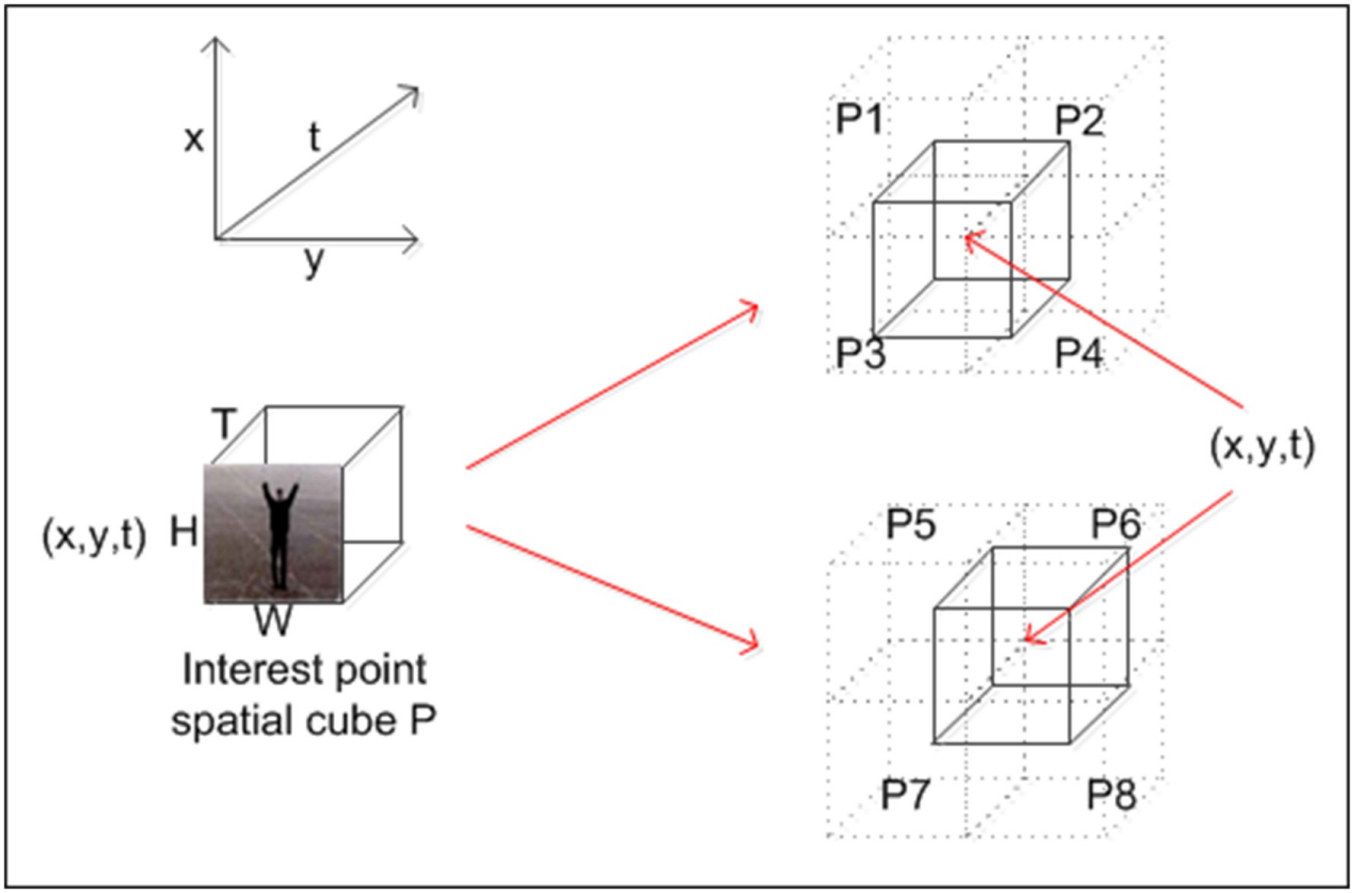
3D HOG and 3D HOF descriptors.

3D HOG and 3D HOF descriptors are used for all detected spatiotemporal interest points to generate the joint feature vector. The method of joint description can be used to obtain the local feature set for training and testing videos, as shown in Fig 3. The process is as follows:

1. Construct a spatial patch *P* using each spatiotemporal interest point (*x*, *y*, *t*) as center. The size of *P* is (*H*, *W*, *T*). Use 3D HOG and 3D HOF feature descriptors to obtain the spatiotemporal feature vector **L**.
2. Use eight vertices of the spatial cube *P* as the centers to construct spatial cubes *P*_1_, *P*_2_, …, *P*_8_ with the same size as *P*, and then use 3D HOG and 3D HOF feature descriptors to obtain the spatiotemporal feature vectors **L**_**1**_, **L**_**2**_, …, **L**_**8**_.
3. Splice the spatiotemporal feature vector **L** and **L**_**1**_, **L**_**2**_, …, **L**_**8**_. That is, the 3D HOG and 3D HOF features of the nine spatial cubes are used as the spatiotemporal descriptor of the interest point (*x*, *y*, *t*).
4. Determine the dimension of the joint descriptor. Usually, a patch contains 18 cells [14, 28]. We chose a 4-bin HOG histogram and a 5-bin HOF histogram. Thus, the corresponding 3D HOG feature dimension is *18* × *4* = *72*, and the 3D HOF feature dimension is *18* × *5* = *90*. The dimension of the joint descriptor of the single patch is *72* + *90* = *162*, and the dimension of the feature vector **L** is *162* × *9* = *1458*.

### Feature fusion

3D HOG and 3D HOF are two types of widely used local feature descriptors, which can accurately describe human action. The extracted HOIRM can be regarded as a middle-level feature between local and global features, which has the advantages of both local and global features, and avoids the complicated steps required for global feature extraction. Accordingly, HOIRM was fused with the local features obtained by the 3D HOG and 3D HOF descriptors to distinguish between different actions more effectively. The extracted 3D HOG, 3D HOF, and HOIRM features are represented in the form of histograms; thus, the features of each frame are integrated by a cumulative histogram as follows:

$$ch(i)=\sum_{i=1}^{n}h(i),$$ (3)

where *ch*(*i*) denotes the i-th interval of the cumulative histogram, *h*(*i*) denotes the i-th interval of the feature histogram, and *n* denotes the number of frames. Then, the 3D HOG, 3D HOF, and HOIRM features can be calculated by Eq (3) and integrated into a feature vector. Then, the final feature vector can be expressed as follows:

$$F=\{ch_{3DHOG},ch_{3DHOF},ch_{HOIRM}\},$$ (4)

where **ch**_**3DHOG**_, **ch**_**3DHOF**_, and **ch**_**HOIRM**_ denote the cumulative histograms of the 3D HOG, 3D HOF, and HOIRM features, respectively.

## Bag of words model based on AP clustering

**Fig 4:**
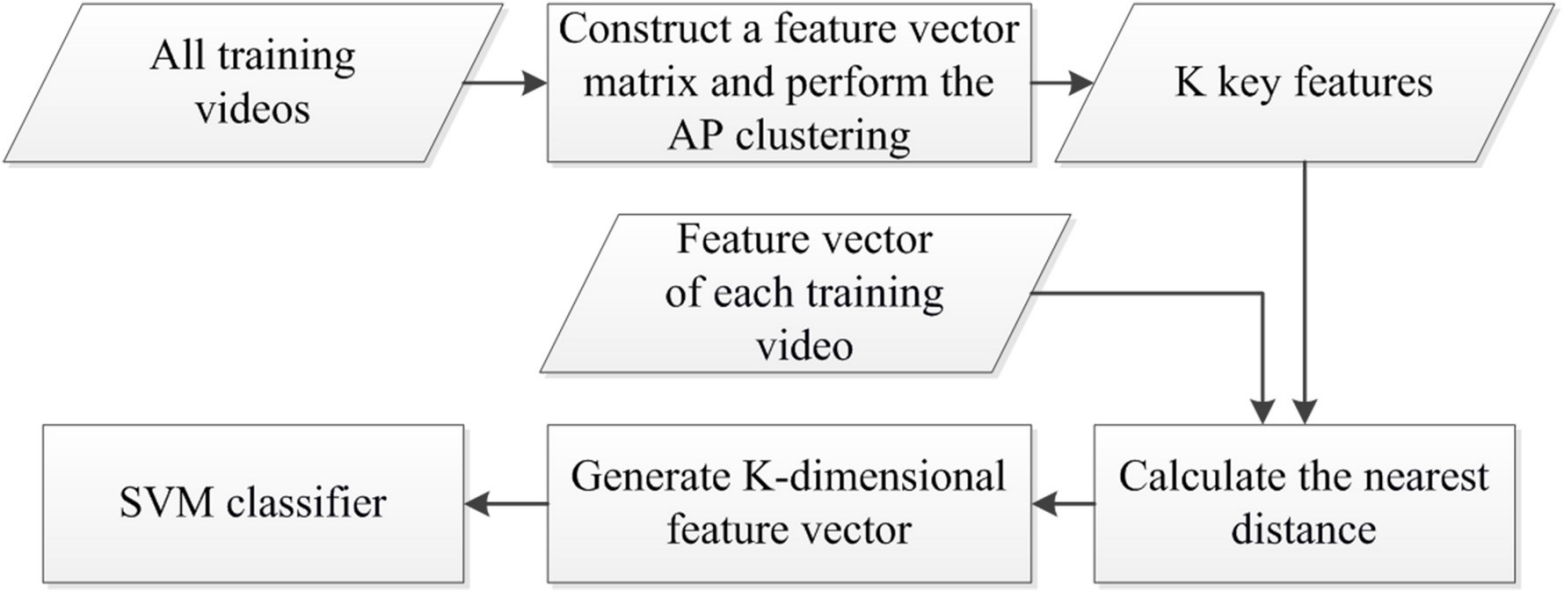
Visual dictionary constructed using the AP clustering BOW algorithm.

In this paper, a BOW model based on the AP clustering algorithm [29] is proposed to overcome the shortcomings of the current BOW model using K-Means clustering in human action recognition. The recognition rate obtained by the current BOW model is usually not high owing to the uncertainty regarding the optimal dictionary capacity. The key idea of the proposed method is to construct a visual dictionary using the AP clustering algorithm for the fused feature vector obtained in the feature extraction stage. The main steps of the method are shown in Fig 4. The implementation includes the following steps:

1. The feature vectors of all training videos are combined to construct a feature vector matrix. Then, AP clustering is performed. If the number of clustering centers is K, a visual dictionary with K words (i.e., K key features) is constructed.
2. A K-dimensional vector is assigned to each training video, and all the components of the vector are initialized to 0, where each component corresponds to a word in the visual dictionary.
3. The distance between the feature vector and K key features is calculated for each training video. Assuming that the distance from the feature vector to the i-th key feature is the nearest, the corresponding i-th component of the K-dimensional vector is set to 1, thus obtaining a K-dimensional feature vector for each training video.
4. The feature vector of each testing video is generated using the same method as in step 3.
5. A support vector machine (SVM) classifier is trained using the training feature vectors obtained in step 3, and the testing video feature vector is classified by the trained SVM to obtain the testing video action category.

## Experimental results and analysis

### Experimental data

#### KTH human action database

The KTH human action database is provided by Schuldt [30] and is currently considered as a standard database for testing action recognition algorithms. It contains a total of 600 videos. Each video is sampled at 25 frames/s, and the spatial resolution of each frame is *160* × *120* pixels. There are six common actions in the database: boxing, hand clapping, hand waving, jogging, running, and walking. Each action is performed by 25 different people in four different scenarios: outdoors, outdoors with scale variation, outdoors with different clothes, and indoors. Although the KTH database contains few action types, it has illumination and scale changes, as well as noise effect and camera jitter in the videos.

#### UCF sports action data set

The UCF sports action data set [31, 32] is a collection of a variety of sport activities from broadcast television channels such as BBC and ESPN. The data set contains 150 video sequences with a resolution of *720* × *480* and 10 sport categories, including diving, Golf swing, kicking, lifting, riding horse, running, skating boarding, swing-bench, swing-side, and walking. The sport backgrounds in the video data set are nature scenes, which are closer to real life. Thus, testing on this database has a more practical significance.

### Evaluation criteria

We used the in-group proportion (IGP) [33] as a measure to evaluate the AP clustering results. IGP is defined as follows: Let *i*^*N*^ denote the closest point to sample *i*, **Class**(*i*) denote the class of sample *i*, and # denote the number of data points satisfying a given condition. Then, for the class denoted by *u*, the IGP measure is defined as

$$\text{IGP}(u)=\frac{\#\{i|\text{Class}(i)=\text{Class}(i^{N})=u\}}{\#\{i|\text{Class}(i)=u\}},$$ (5)

where #{*i* | **Class**(*i*) = **Class**(*i*^*N*^) = *u*} denotes the number of data points that are closest to sample *i* and belong to the same class as sample *i*(i.e., *u*). #{*i* | **Class**(*i*) = *u*} denotes the total number of data points in the class *u*. Clearly, a higher IGP value indicates better clustering. For the same IGP value, the largest number of categories should be selected as the best number of clusters.

### Results and analysis

#### KTH database experimental results

**Fig 5:**
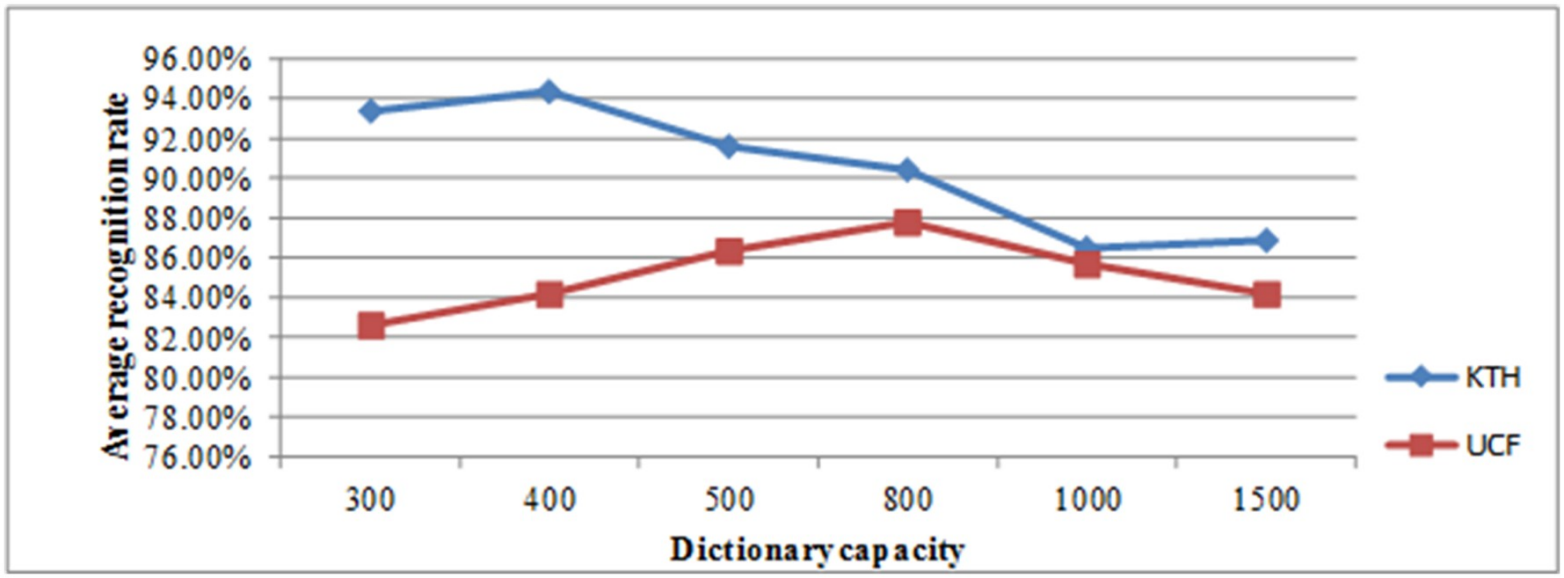
Change in the average recognition rate in the KTH and UCF databases under different dictionary capacities.

**Fig 6:**
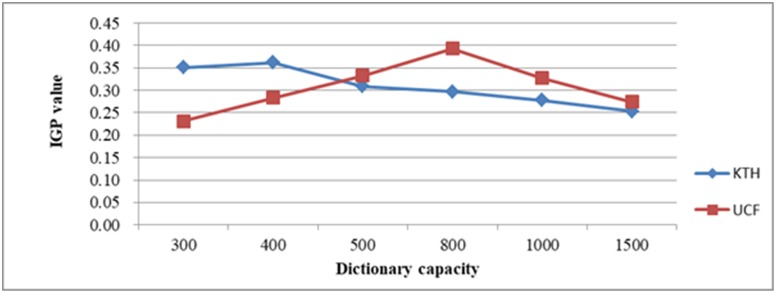
Change in the IGP value in the KTH and UCF databases under different dictionary capacities.

First, we experimented with all the videos in the KTH database and constructed a visual dictionary using a BOW model based on K-Means clustering. We used the fused features by the proposed method. We set the capacity of the visual dictionary to 300, 400, 500, 800, 1000, and 1500, and used leave-one-out cross validation. In the KTH database, each action class contains 100 videos. For each action class, we randomly took 80 videos as the training set, and the remaining 20 videos were taken as the testing set. We conducted five experiments for each action class and calculated the average recognition rate of the experiments. Thereafter, we calculated the mean of the six action class recognition rates as the average recognition rate of the entire database under a certain dictionary capacity. Therefore, for a certain dictionary capacity, the experiments required 30 training and testing runs, and for the six different dictionary capacities, 180 training and testing runs were required. The recognition rate in the KTH database under different dictionary capacities is shown in Table 1. After setting the visual dictionary capacity, the IGP measure was calculated. The experimental results regarding the action recognition rate, the IGP value, and running time under different dictionary capacities are shown in Table 2. Figs 5 and 6 show the change in the average recognition rate and the IGP value, respectively, under different dictionary capacities.

**Table 1:** Recognition rate in the KTH database under different dictionary capacities (%).

Dictionary capacity	300	400	500	800	1000	1500
Boxing	100.00	100.00	100.00	98.55	95.20	95.60
Hand clapping	94.33	95.00	94.50	94.25	87.33	88.25
Hand waving	98.75	100.00	97.33	95.50	90.00	90.00
Jogging	84.33	85.50	85.50	86.50	80.50	78.50
Running	88.25	90.00	80.50	80.33	80.50	82.33
Walking	94.50	95.50	92.00	87.23	85.50	86.50
Average	93.36	94.33	91.63	90.39	86.50	86.86

**Table 2:** Experimental results of BOW based on K-Means clustering and AP clustering in the KTH database.

Clustering method	Visual dictionary capacity	Average recognition rate (%)	IGP value	Running time (s)
K-MEANS	300	93.36	0.3506	28644.5
	400	94.33	0.3617	35748.9
	500	91.63	0.3089	42132.1
	800	90.39	0.2974	49628.5
	1000	86.50	0.2776	53125.1
	1500	86.86	0.2535	65535.1
	**379**	**95.10**	**0.3598**	**30878.2**
**AP**	**379**	**95.75**	**0.4145**	**10576.3**

It can be seen from Tables 1 and 2 and Figs 5 and 6 that the average recognition rate and the IGP first increase as the dictionary capacity increases from 300 to 400, and then decrease as the dictionary capacity increases from 400 to 1500. The recognition rate and the IGP are the highest when the dictionary capacity is 400, which implies that the best dictionary capacity should be approximately 400. However, the determination of the exact optimal dictionary capacity requires more experiments.

In the above experiments, the most difficult problem was that the optimal dictionary capacity could not be determined. Therefore, we could only obtain the recognition rate under different dictionary capacities through several experiments, thereby obtaining a better but not the optimal rate. To obtain the optimal action recognition rate (on the basis of multi-feature fusion) we used the AP clustering algorithm to calculate the visual dictionary capacity; the results are shown in Table 2. The obtained capacity is 379 and the IGP is 0.4145, which is obviously larger than the IGP obtained by the K-Means clustering algorithm. The recognition rate is also increased to 95.75%, which is higher than that obtained by the K-Means clustering algorithm. Moreover, the BOW modeling method based on AP clustering requires only a single calculation to obtain the best dictionary capacity, and the running time is also smaller than that of the method based on K-Means clustering.

Then, we set the dictionary capacity of the K-Means clustering algorithm to 379 to verify the clustering effect of the AP clustering algorithm on the fused feature. The recognition rate, IGP, and running time of the two clustering algorithms are compared for the same dictionary capacity. The results are also shown in Table 2. It can be seen that for the same dictionary capacity, the IGP of the K-Means clustering algorithm is smaller than that of the AP clustering algorithm. The recognition rate obtained by the K-Means clustering algorithm is 95.10%, which is lower than that by the AP clustering algorithm (95.75%). The running time of the AP clustering algorithm is considerably lower than that of the K-Means clustering algorithm. Therefore, the clustering effect of the AP clustering algorithm is better than that of the K-Means clustering algorithm after multi-feature fusion.

#### UCF database experimental results

For the UCF database, we also set the capacity of the visual dictionary to 300, 400, 500, 800, 1000, and 1500. The results for the action recognition rate and the IGP using feature fusion are shown in Tables 3 and 4 and Figs 5 and 6. The average recognition rate and the IGP increase as the dictionary capacity increases from 300 to 800, and decrease as the dictionary capacity increases from 800 to 1500, which implies that the best dictionary capacity should be approximately 800. However, the determination of the exact optimal cluster number requires more experiments.

**Table 3:** Recognition rate in the UCF data set under different dictionary capacities (%).

Dictionary capacity	300	400	500	800	1000	1500
Diving	95.80	96.50	100.00	100.00	98.00	96.50
Golf swing	84.80	85.50	86.80	87.60	86.80	85.50
Kicking	87.80	88.00	89.80	91.50	90.00	88.00
Lifting	70.20	71.80	74.50	75.80	72.10	71.80
Riding horse	65.20	67.60	69.50	70.80	70.60	67.60
Running	70.00	74.20	76.10	78.80	75.20	74.20
Skating boarding	83.20	85.00	86.80	88.50	86.40	85.00
Swing-Bench	90.00	91.50	92.10	93.50	90.50	91.50
Swing-Side	94.80	95.20	98.00	100.00	98.80	95.20
Walking	84.30	86.50	90.00	91.30	88.80	86.50
Average	82.61	84.18	86.36	87.78	85.72	84.18

**Table 4:** Experimental results of BOW based on K-Means clustering and AP clustering in the UCF data set.

Clustering method	Visual dictionary capacity	Average recognition rate (%)	IGP value	Running time (s)
K-Means	300	82.61	0.2314	20248.1
	400	84.98	0.2836	27483.5
	500	86.36	0.3325	31320.2
	800	87.78	0.3928	38288.5
	1000	85.72	0.3275	40749.9
	1500	84.18	0.2743	52735.8
	**758**	**87.95**	**0.4058**	**35659.4**
AP	**758**	**88.25**	**0.4835**	**14457.3**

Then, we constructed a visual dictionary by the AP clustering algorithm and obtained the dictionary capacity, the IGP, and the corresponding recognition rate, which are shown in Table 4. The IGP value and the recognition rate are both higher than those obtained by the K-Means clustering algorithm, and the running time of a single experiment is also lower than that by the K-Means clustering algorithm. These results are similar to the results for the KTH database, further demonstrating that AP clustering is more effective and faster than K-Means clustering.

Table 4 also shows the recognition rate, the IGP, and the running time obtained by the two clustering algorithms under the same dictionary capacity using the UCF data sets. If the dictionary capacity is 758, the IGP and the recognition rate obtained by the K-Means clustering algorithm are smaller than those by the AP clustering algorithm. Moreover, the running time of the K-Means clustering algorithm is higher than that of the AP clustering algorithm. Therefore, the AP clustering algorithm is more effective than the K-Means clustering algorithm.

#### Comparative experiments for different features

The above experimental results demonstrate that using the AP clustering algorithm can effectively reduce the number of experiments and the BOW model construction time. Furthermore, a reasonable visual dictionary capacity is obtained to improve the recognition rate. Accordingly, the AP clustering algorithm was used to compare the recognition performance of a variety of features. In the experiments, 3D HOG combined with 3D HOF (3D HOG+3D HOF), HOIRM, and the proposed fused features were used for action recognition in the two databases. The results are shown in Table 5. For both datasets, the recognition rate obtained using HOIRM is higher than that obtained by 3D HOG combined with 3D HOF, and the recognition rate obtained by the fused features is the highest. Thus, the proposed fused feature is superior to 3D HOG+3D HOF or HOIRM for action recognition.

**Table 5:** Comparison of recognition rates of three different features (%).

Features	KTH database	UCF dataset
3D HOG+3D HOF	91.50	85.95
HOIRM	92.43	86.52
The proposed fused feature	95.75	88.25

#### Comparative experiments for different methods

Table 6 compares the recognition rate of the proposed method with that of some other methods. The average recognition rate of the proposed method is 95.75% in the KTH database, and 88.25% in the UCF database, which are both higher than the recognition rate obtained by other methods. The two databases are both close to real scenes. There are illumination and scale changes, as well as noise and camera jitter in the KTH database, and the UCF database has various types of actions in complex scenes. Therefore, the superior performance of the proposed method in those two databases demonstrates that this method can improve the recognition rate of human action in real scenes and has high feasibility and robustness.

**Table 6:** Comparison of the recognition rate of the proposed method with that of other methods.

KTH database		UCF dataset	
Methods	recognition rate (%)	Methods	recognition rate (%)
Naidoo [11] method	82.00	Wang [20] method	85.60
Jaouedi [12] method	91.00	Klaser [21] method	86.70
Zhang [13] method	91.67	Bregonizo [22] method	86.90
Laptev [14] method	91.80	Farrajota[23] method	87.20
Najar [15] method	91.97	The proposed method	88.25
Yuan [16] method	93.30		
Tong [17] method	93.96		
Fu [18] method	94.33		
Kovashka [19] method	94.53		
The proposed method	95.75		

## Conclusion

In this paper, the HOIRM feature was proposed and fused with 3D HOG and 3D HOF features near the spatiotemporal interest points for action recognition. The experimental results demonstrated that the proposed fused feature can improve the performance of human action recognition and obtain a higher recognition rate compared to 3D HOG+3D HOF or HOIRM. Moreover, we used the AP clustering algorithm to construct a BOW model. The experimental results demonstrated that the AP clustering algorithm can effectively reduce the number of experiments and the model construction time; furthermore, a reasonable visual dictionary capacity is automatically obtained to improve the recognition rate. Compared with some other methods, the proposed method obtained higher average recognition rate in the KTH and UCF databases.

Future work will focus on the following: (1) Further research should be conducted on the AP clustering algorithm and adjustment of its parameters to obtain better visual dictionary capacity and hence better action recognition rate. (2) The present method is suitable only for single-person single-action recognition. Thus, it should be extended to multi-person interactive action. (3) The present method should be further improved so that it may be more robust for real-scene action, which is often irregular and discontinuous.

## Supporting information

S1 File Certificate of editing. (PDF)

S2 File Polished manuscript by Editage. (DOC)
